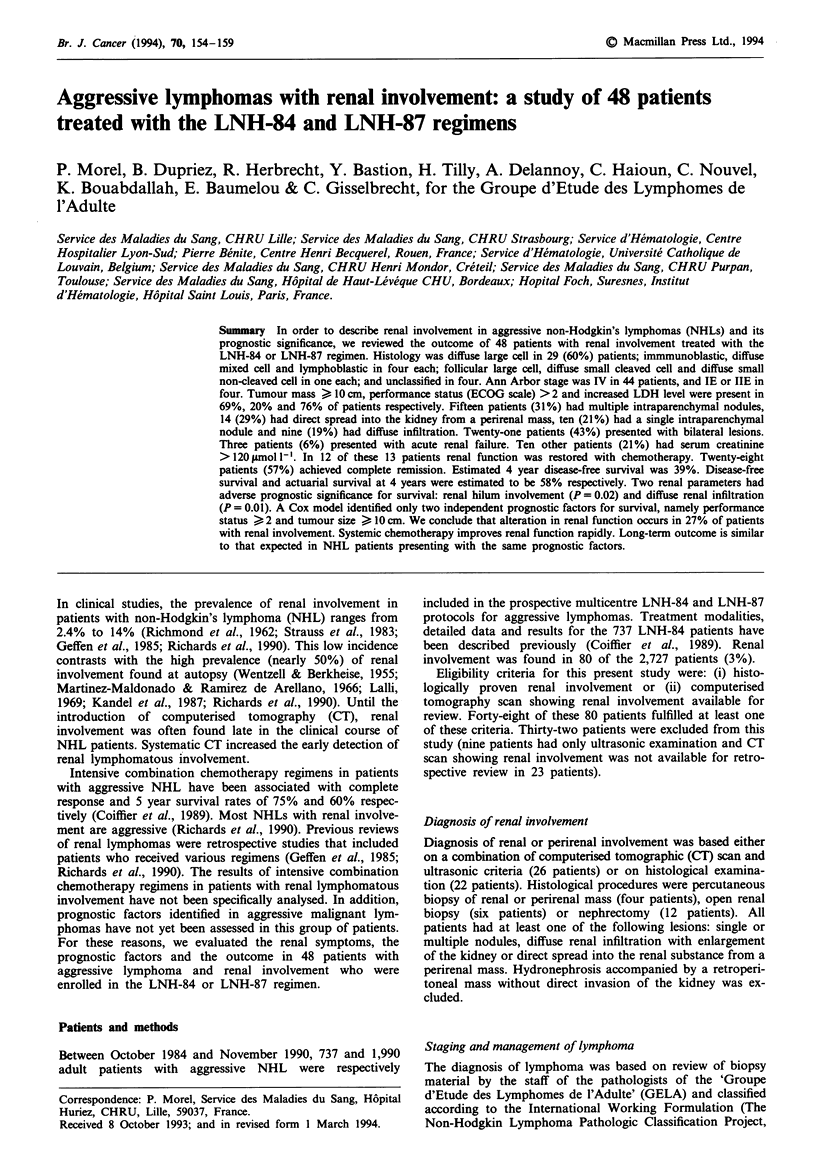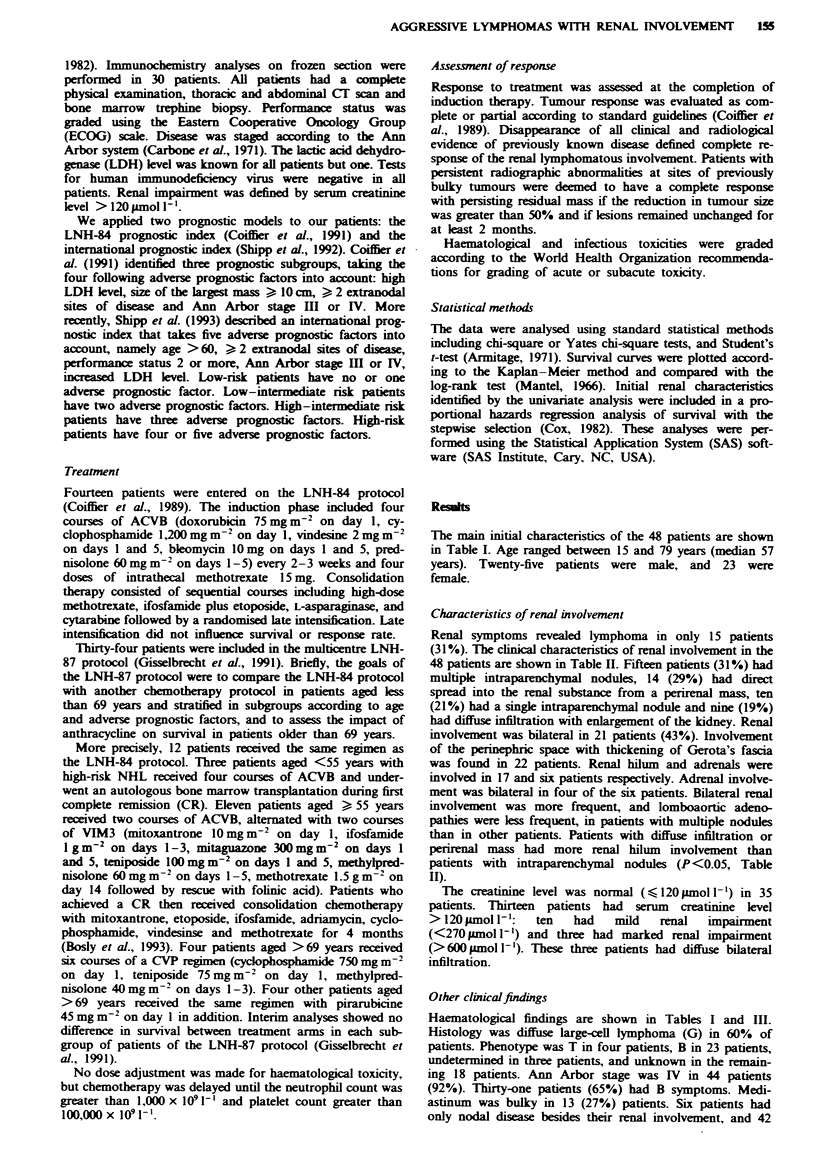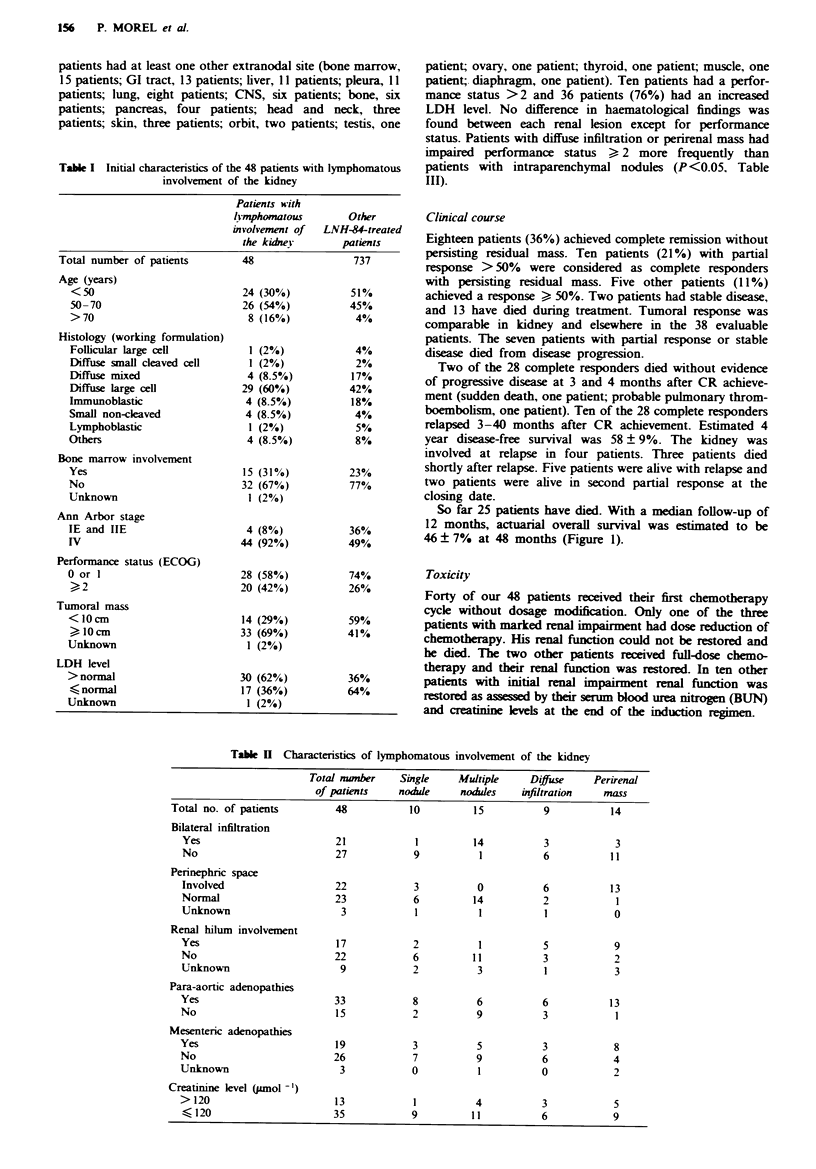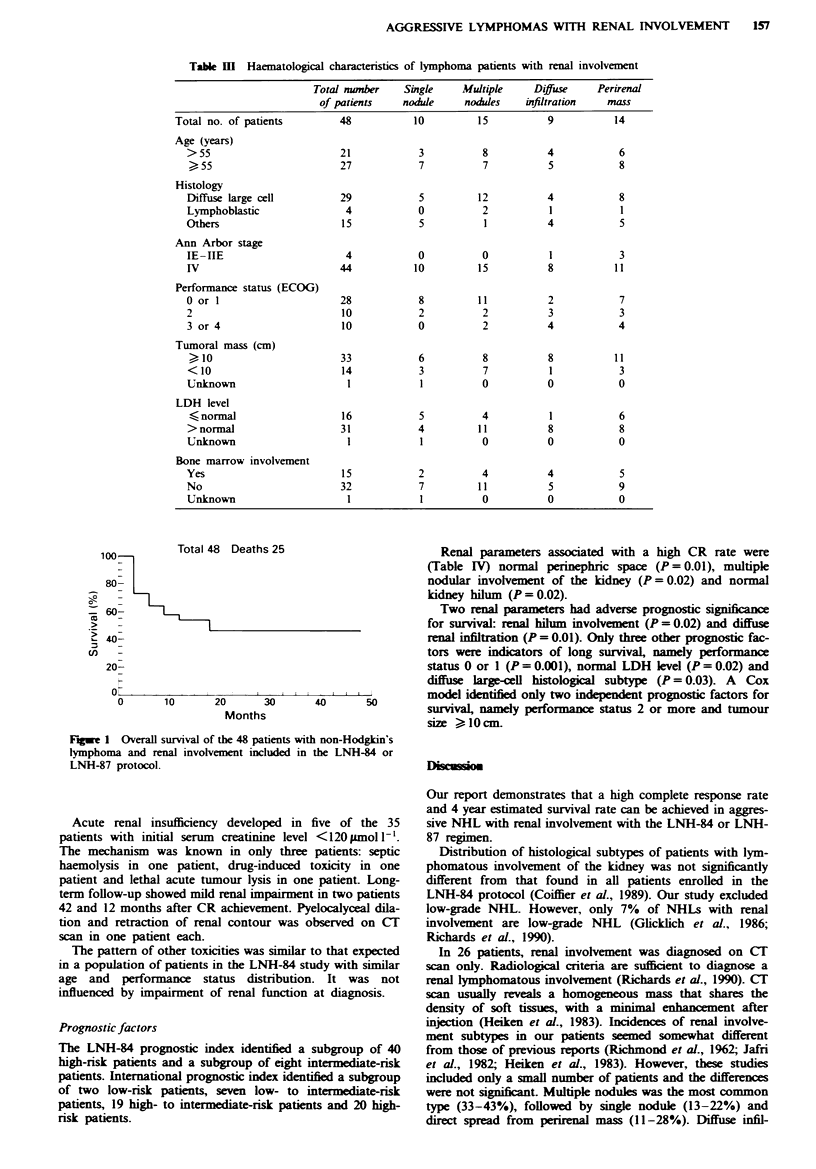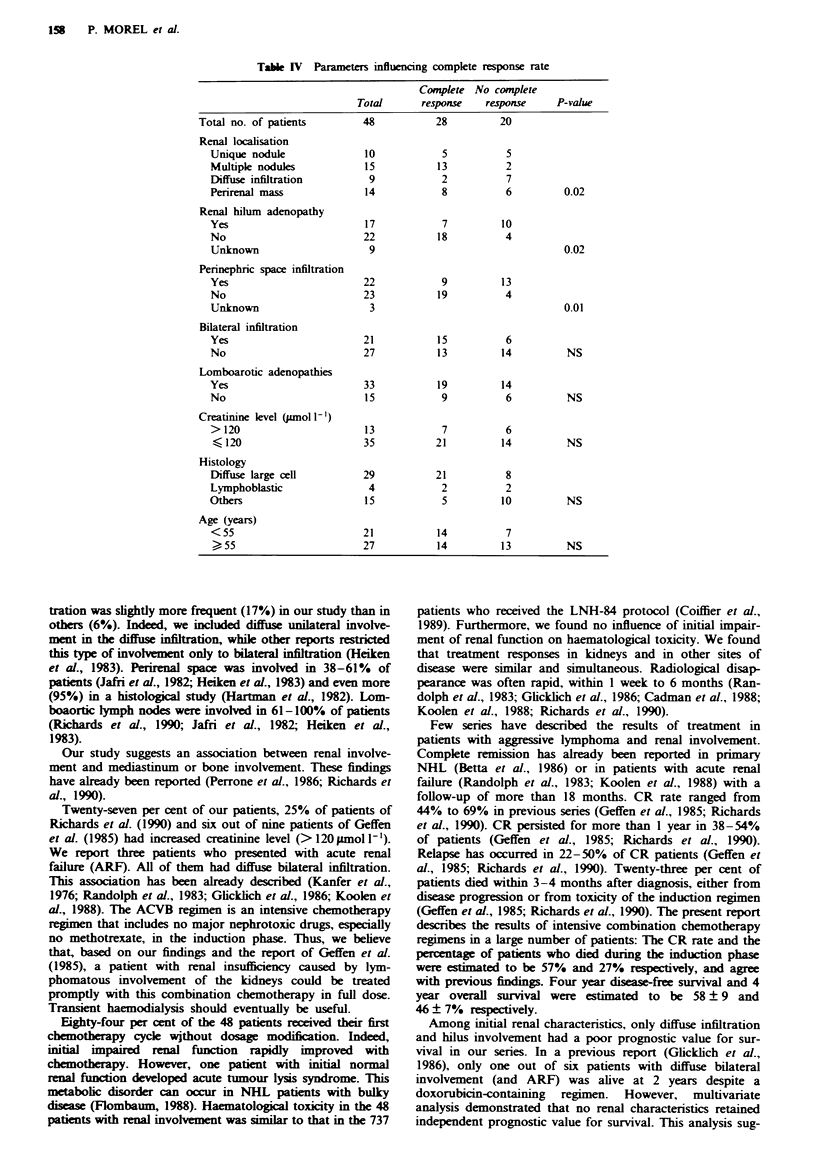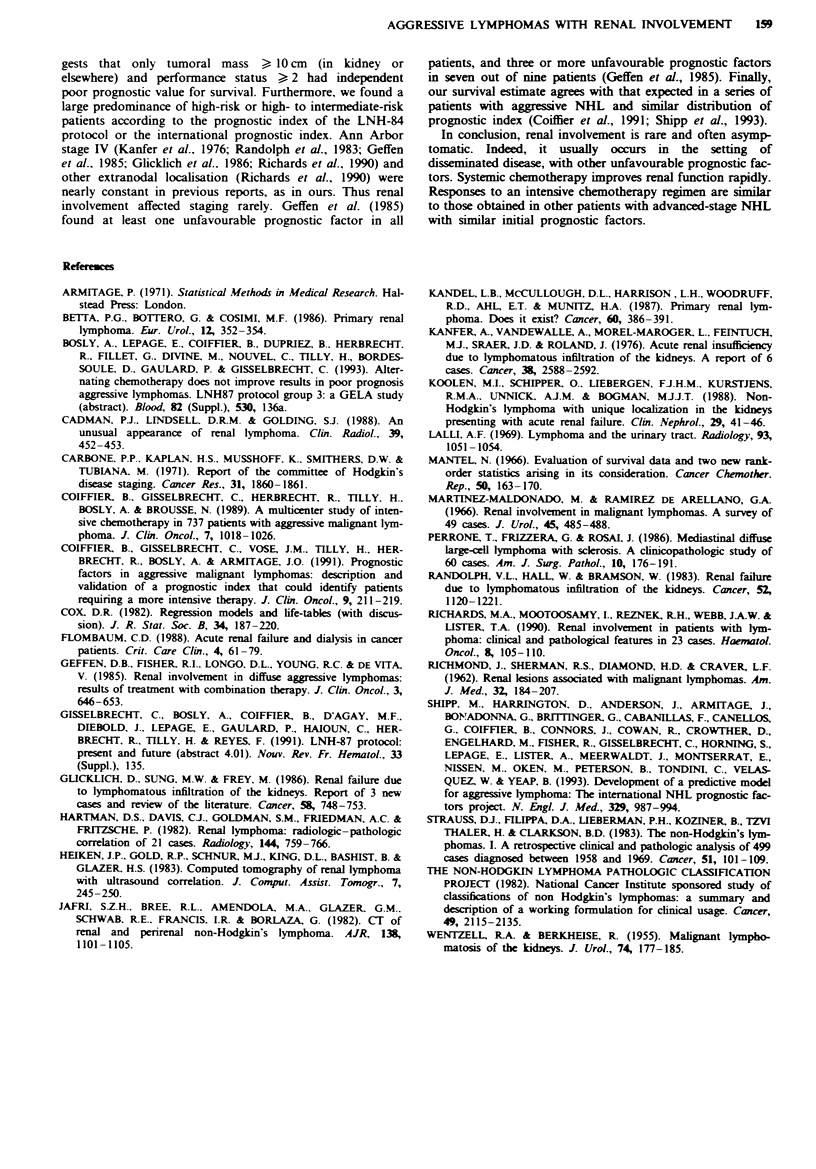# Aggressive lymphomas with renal involvement: a study of 48 patients treated with the LNH-84 and LNH-87 regimens. Groupe d'Etude des Lymphomes de l'Adulte.

**DOI:** 10.1038/bjc.1994.267

**Published:** 1994-07

**Authors:** P. Morel, B. Dupriez, R. Herbrecht, Y. Bastion, H. Tilly, A. Delannoy, C. Haioun, C. Nouvel, K. Bouabdallah, E. Baumelou

**Affiliations:** Service des Maladies du Sang, CHRU Lille, France.

## Abstract

In order to describe renal involvement in aggressive non-Hodgkin's lymphomas (NHLs) and its prognostic significance, we reviewed the outcome of 48 patients with renal involvement treated with the LNH-84 or LNH-87 regimen. Histology was diffuse large cell in 29 (60%) patients; immunoblastic, diffuse mixed cell and lymphoblastic in four each; follicular large cell, diffuse small cleaved cell and diffuse small non-cleaved cell in one each; and unclassified in four. Ann Arbor stage was IV in 44 patients, and IE or IIE in four. Tumour mass > or = 10 cm, performance status (ECOG scale) > 2 and increased LDH level were present in 69%, 20% and 76% of patients respectively. Fifteen patients (31%) had multiple intraparenchymal nodules, 14 (29%) had direct spread into the kidney from a perirenal mass, ten (21%) had a single intraparenchymal nodule and nine (19%) had diffuse infiltration. Twenty-one patients (43%) presented with bilateral lesions. Three patients (6%) presented with acute renal failure. Ten other patients (21%) had serum creatinine > 120 mumol l-1. In 12 of these 13 patients renal function was restored with chemotherapy. Twenty-eight patients (57%) achieved complete remission. Estimated 4 year disease-free survival was 39%. Disease-free survival and actuarial survival at 4 years were estimated to be 58% respectively. Two renal parameters had adverse prognostic significance for survival: renal hilum involvement (P = 0.02) and diffuse renal infiltration (P = 0.01). A Cox model identified only two independent prognostic factors for survival, namely performance status > or = 2 and tumour size > or = 10 cm. We conclude that alteration in renal function occurs in 27% of patients with renal involvement. Systemic chemotherapy improves renal function rapidly. Long-term outcome is similar to that expected in NHL patients presenting with the same prognostic factors.


Br. J. Cancer (1994), 70, 154-159 © Macmillan Press Ltd., 1994

### Aggressive lymphomas with renal involvement: a study of 48 patients treated with the LNH-84 and LNH-87 regimens

P. Morel, B. Dupriez, R. Herbrecht, Y. Bastion, H. Tilly, A. Delannoy, C. Haioun, C. Nouvel, K. Bouabdallah, E. Baumelou & C. Gisselbrecht, for the Groupe d'Etude des Lymphomes de l'Adulte

*Service des Maladies du Sang, CHRU Lille; Service des Maladies du Sang, CHRU Strasbourg; Service d'Hématologie, Centre Hospitalier Lyon-Sud; Pierre Bénite, Centre Henri Becquerel, Rouen, France; Service d'Hématologie, Université Catholique de Louvain, Belgium; Service des Maladies du Sang, CHRU Henri Mondor, Créteil; Service des Maladies du Sang, CHRU Purpan, Toulouse; Service des Maladies du Sang, Hôpital de Haut-Lévêque CHU, Bordeaux; Hopital Foch, Suresnes, Institut d'Hématologie, Hôpital Saint Louis, Paris, France.*

**Summary** In order to describe renal involvement in aggressive non-Hodgkin's lymphomas (NHLs) and its prognostic significance, we reviewed the outcome of 48 patients with renal involvement treated with the LNH-84 or LNH-87 regimen. Histology was diffuse large cell in 29 (60%) patients; immmunoblastic, diffuse mixed cell and lymphoblastic in four each; follicular large cell, diffuse small cleaved cell and diffuse small non-cleaved cell in one each; and unclassified in four. Ann Arbor stage was IV in 44 patients, and IE or IIE in four. Tumour mass $\geqslant$ 10 cm, performance status (ECOG scale) > 2 and increased LDH level were present in 69%, 20% and 76% of patients respectively. Fifteen patients (31%) had multiple intraparenchymal nodules, 14 (29%) had direct spread into the kidney from a perirenal mass, ten (21%) had a single intraparenchymal nodule and nine (19%) had diffuse infiltration. Twenty-one patients (43%) presented with bilateral lesions. Three patients (6%) presented with acute renal failure. Ten other patients (21%) had serum creatinine > 120 $\mu mol\ l^{-1}$. In 12 of these 13 patients renal function was restored with chemotherapy. Twenty-eight patients (57%) achieved complete remission. Estimated 4 year disease-free survival was 39%. Disease-free survival and actuarial survival at 4 years were estimated to be 58% respectively. Two renal parameters had adverse prognostic significance for survival: renal hilum involvement ($P = 0.02$) and diffuse renal infiltration ($P = 0.01$). A Cox model identified only two independent prognostic factors for survival, namely performance status $\geqslant$ 2 and tumour size $\geqslant$ 10 cm. We conclude that alteration in renal function occurs in 27% of patients with renal involvement. Systemic chemotherapy improves renal function rapidly. Long-term outcome is similar to that expected in NHL patients presenting with the same prognostic factors.

In clinical studies, the prevalence of renal involvement in patients with non-Hodgkin's lymphoma (NHL) ranges from 2.4% to 14% (Richmond *et al.*, 1962; Strauss *et al.*, 1983; Geffen *et al.*, 1985; Richards *et al.*, 1990). This low incidence contrasts with the high prevalence (nearly 50%) of renal involvement found at autopsy (Wentzell & Berkheise, 1955; Martinez-Maldonado & Ramirez de Arellano, 1966; Lalli, 1969; Kandel *et al.*, 1987; Richards *et al.*, 1990). Until the introduction of computerised tomography (CT), renal involvement was often found late in the clinical course of NHL patients. Systematic CT increased the early detection of renal lymphomatous involvement.

Intensive combination chemotherapy regimens in patients with aggressive NHL have been associated with complete response and 5 year survival rates of 75% and 60% respectively (Coiffier *et al.*, 1989). Most NHLs with renal involvement are aggressive (Richards *et al.*, 1990). Previous reviews of renal lymphomas were retrospective studies that included patients who received various regimens (Geffen *et al.*, 1985; Richards *et al.*, 1990). The results of intensive combination chemotherapy regimens in patients with renal lymphomatous involvement have not been specifically analysed. In addition, prognostic factors identified in aggressive malignant lymphomas have not yet been assessed in this group of patients. For these reasons, we evaluated the renal symptoms, the prognostic factors and the outcome in 48 patients with aggressive lymphoma and renal involvement who were enrolled in the LNH-84 or LNH-87 regimen.

### Patients and methods

Between October 1984 and November 1990, 737 and 1,990 adult patients with aggressive NHL were respectively included in the prospective multicentre LNH-84 and LNH-87 protocols for aggressive lymphomas. Treatment modalities, detailed data and results for the 737 LNH-84 patients have been described previously (Coiffier *et al.*, 1989). Renal involvement was found in 80 of the 2,727 patients (3%).

Eligibility criteria for this present study were: (i) histologically proven renal involvement or (ii) computerised tomography scan showing renal involvement available for review. Forty-eight of these 80 patients fulfilled at least one of these criteria. Thirty-two patients were excluded from this study (nine patients had only ultrasonic examination and CT scan showing renal involvement was not available for retrospective review in 23 patients).

### *Diagnosis of renal involvement*

Diagnosis of renal or perirenal involvement was based either on a combination of computerised tomographic (CT) scan and ultrasonic criteria (26 patients) or on histological examination (22 patients). Histological procedures were percutaneous biopsy of renal or perirenal mass (four patients), open renal biopsy (six patients) or nephrectomy (12 patients). All patients had at least one of the following lesions: single or multiple nodules, diffuse renal infiltration with enlargement of the kidney or direct spread into the renal substance from a perirenal mass. Hydronephrosis accompanied by a retroperitoneal mass without direct invasion of the kidney was excluded.

### *Staging and management of lymphoma*

The diagnosis of lymphoma was based on review of biopsy material by the staff of the pathologists of the 'Groupe d'Etude des Lymphomes de l'Adulte' (GELA) and classified according to the International Working Formulation (The Non-Hodgkin Lymphoma Pathologic Classification Project,

Correspondence: P. Morel, Service des Maladies du Sang, Hôpital Huriez, CHRU, Lille, 59037, France.
Received 8 October 1993; and in revised form 1 March 1994.

1982). Immunochemistry analyses on frozen section were performed in 30 patients. All patients had a complete physical examination, thoracic and abdominal CT scan and bone marrow trephine biopsy. Performance status was graded using the Eastern Cooperative Oncology Group (ECOG) scale. Disease was staged according to the Ann Arbor system (Carbone *et al.*, 1971). The lactic acid dehydrogenase (LDH) level was known for all patients but one. Tests for human immunodeficiency virus were negative in all patients. Renal impairment was defined by serum creatinine level $>120\,\mu mol\,l^{-1}$.

We applied two prognostic models to our patients: the LNH-84 prognostic index (Coiffier *et al.*, 1991) and the international prognostic index (Shipp *et al.*, 1992). Coiffier *et al.* (1991) identified three prognostic subgroups, taking the four following adverse prognostic factors into account: high LDH level, size of the largest mass $\geqslant 10$ cm, $\geqslant 2$ extranodal sites of disease and Ann Arbor stage III or IV. More recently, Shipp *et al.* (1993) described an international prognostic index that takes five adverse prognostic factors into account, namely age $>60$, $\geqslant 2$ extranodal sites of disease, performance status 2 or more, Ann Arbor stage III or IV, increased LDH level. Low-risk patients have no or one adverse prognostic factor. Low–intermediate risk patients have two adverse prognostic factors. High–intermediate risk patients have three adverse prognostic factors. High-risk patients have four or five adverse prognostic factors.

### *Treatment*

Fourteen patients were entered on the LNH-84 protocol (Coiffier *et al.*, 1989). The induction phase included four courses of ACVB (doxorubicin $75\,mg\,m^{-2}$ on day 1, cyclophosphamide $1{,}200\,mg\,m^{-2}$ on day 1, vindesine $2\,mg\,m^{-2}$ on days 1 and 5, bleomycin 10 mg on days 1 and 5, prednisolone $60\,mg\,m^{-2}$ on days 1–5) every 2–3 weeks and four doses of intrathecal methotrexate 15 mg. Consolidation therapy consisted of sequential courses including high-dose methotrexate, ifosfamide plus etoposide, L-asparaginase, and cytarabine followed by a randomised late intensification. Late intensification did not influence survival or response rate.

Thirty-four patients were included in the multicentre LNH-87 protocol (Gisselbrecht *et al.*, 1991). Briefly, the goals of the LNH-87 protocol were to compare the LNH-84 protocol with another chemotherapy protocol in patients aged less than 69 years and stratified in subgroups according to age and adverse prognostic factors, and to assess the impact of anthracycline on survival in patients older than 69 years.

More precisely, 12 patients received the same regimen as the LNH-84 protocol. Three patients aged $<55$ years with high-risk NHL received four courses of ACVB and underwent an autologous bone marrow transplantation during first complete remission (CR). Eleven patients aged $\geqslant 55$ years received two courses of ACVB, alternated with two courses of VIM3 (mitoxantrone $10\,mg\,m^{-2}$ on day 1, ifosfamide $1\,g\,m^{-2}$ on days 1–3, mitaguazone $300\,mg\,m^{-2}$ on days 1 and 5, teniposide $100\,mg\,m^{-2}$ on days 1 and 5, methylprednisolone $60\,mg\,m^{-2}$ on days 1–5, methotrexate $1.5\,g\,m^{-2}$ on day 14 followed by rescue with folinic acid). Patients who achieved a CR then received consolidation chemotherapy with mitoxantrone, etoposide, ifosfamide, adriamycin, cyclophosphamide, vindesinse and methotrexate for 4 months (Bosly *et al.*, 1993). Four patients aged $>69$ years received six courses of a CVP regimen (cyclophosphamide $750\,mg\,m^{-2}$ on day 1, teniposide $75\,mg\,m^{-2}$ on day 1, methylprednisolone $40\,mg\,m^{-2}$ on days 1–3). Four other patients aged $>69$ years received the same regimen with pirarubicine $45\,mg\,m^{-2}$ on day 1 in addition. Interim analyses showed no difference in survival between treatment arms in each subgroup of patients of the LNH-87 protocol (Gisselbrecht *et al.*, 1991).

No dose adjustment was made for haematological toxicity, but chemotherapy was delayed until the neutrophil count was greater than $1{,}000 \times 10^9\,l^{-1}$ and platelet count greater than $100{,}000 \times 10^9\,l^{-1}$.

### *Assessment of response*

Response to treatment was assessed at the completion of induction therapy. Tumour response was evaluated as complete or partial according to standard guidelines (Coiffier *et al.*, 1989). Disappearance of all clinical and radiological evidence of previously known disease defined complete response of the renal lymphomatous involvement. Patients with persistent radiographic abnormalities at sites of previously bulky tumours were deemed to have a complete response with persisting residual mass if the reduction in tumour size was greater than 50% and if lesions remained unchanged for at least 2 months.

Haematological and infectious toxicities were graded according to the World Health Organization recommendations for grading of acute or subacute toxicity.

### *Statistical methods*

The data were analysed using standard statistical methods including chi-square or Yates chi-square tests, and Student's *t*-test (Armitage, 1971). Survival curves were plotted according to the Kaplan–Meier method and compared with the log-rank test (Mantel, 1966). Initial renal characteristics identified by the univariate analysis were included in a proportional hazards regression analysis of survival with the stepwise selection (Cox, 1982). These analyses were performed using the Statistical Application System (SAS) software (SAS Institute, Cary, NC, USA).

### Results

The main initial characteristics of the 48 patients are shown in Table I. Age ranged between 15 and 79 years (median 57 years). Twenty-five patients were male, and 23 were female.

### *Characteristics of renal involvement*

Renal symptoms revealed lymphoma in only 15 patients (31%). The clinical characteristics of renal involvement in the 48 patients are shown in Table II. Fifteen patients (31%) had multiple intraparenchymal nodules, 14 (29%) had direct spread into the renal substance from a perirenal mass, ten (21%) had a single intraparenchymal nodule and nine (19%) had diffuse infiltration with enlargement of the kidney. Renal involvement was bilateral in 21 patients (43%). Involvement of the perinephric space with thickening of Gerota's fascia was found in 22 patients. Renal hilum and adrenals were involved in 17 and six patients respectively. Adrenal involvement was bilateral in four of the six patients. Bilateral renal involvement was more frequent, and lomboaortic adenopathies were less frequent, in patients with multiple nodules than in other patients. Patients with diffuse infiltration or perirenal mass had more renal hilum involvement than patients with intraparenchymal nodules ($P<0.05$, Table II).

The creatinine level was normal ($\leqslant 120\,\mu mol\,l^{-1}$) in 35 patients. Thirteen patients had serum creatinine level $>120\,\mu mol\,l^{-1}$: ten had mild renal impairment ($<270\,\mu mol\,l^{-1}$) and three had marked renal impairment ($>600\,\mu mol\,l^{-1}$). These three patients had diffuse bilateral infiltration.

### *Other clinical findings*

Haematological findings are shown in Tables I and III. Histology was diffuse large-cell lymphoma (G) in 60% of patients. Phenotype was T in four patients, B in 23 patients, undetermined in three patients, and unknown in the remaining 18 patients. Ann Arbor stage was IV in 44 patients (92%). Thirty-one patients (65%) had B symptoms. Mediastinum was bulky in 13 (27%) patients. Six patients had only nodal disease besides their renal involvement, and 42

patients had at least one other extranodal site (bone marrow, 15 patients; GI tract, 13 patients; liver, 11 patients; pleura, 11 patients; lung, eight patients; CNS, six patients; bone, six patients; pancreas, four patients; head and neck, three patients; skin, three patients; orbit, two patients; testis, one patient; ovary, one patient; thyroid, one patient; muscle, one patient; diaphragm, one patient). Ten patients had a performance status >2 and 36 patients (76%) had an increased LDH level. No difference in haematological findings was found between each renal lesion except for performance status. Patients with diffuse infiltration or perirenal mass had impaired performance status ⩾2 more frequently than patients with intraparenchymal nodules ($P<0.05$, Table III).

**Table I** Initial characteristics of the 48 patients with lymphomatous involvement of the kidney

| | *Patients with lymphomatous involvement of the kidney* | *Other LNH-84-treated patients* |
|---|---|---|
| Total number of patients | 48 | 737 |
| Age (years) | | |
| <50 | 24 (30%) | 51% |
| 50–70 | 26 (54%) | 45% |
| >70 | 8 (16%) | 4% |
| Histology (working formulation) | | |
| Follicular large cell | 1 (2%) | 4% |
| Diffuse small cleaved cell | 1 (2%) | 2% |
| Diffuse mixed | 4 (8.5%) | 17% |
| Diffuse large cell | 29 (60%) | 42% |
| Immunoblastic | 4 (8.5%) | 18% |
| Small non-cleaved | 4 (8.5%) | 4% |
| Lymphoblastic | 1 (2%) | 5% |
| Others | 4 (8.5%) | 8% |
| Bone marrow involvement | | |
| Yes | 15 (31%) | 23% |
| No | 32 (67%) | 77% |
| Unknown | 1 (2%) | |
| Ann Arbor stage | | |
| IE and IIE | 4 (8%) | 36% |
| IV | 44 (92%) | 49% |
| Performance status (ECOG) | | |
| 0 or 1 | 28 (58%) | 74% |
| ⩾2 | 20 (42%) | 26% |
| Tumoral mass | | |
| <10 cm | 14 (29%) | 59% |
| ⩾10 cm | 33 (69%) | 41% |
| Unknown | 1 (2%) | |
| LDH level | | |
| >normal | 30 (62%) | 36% |
| ⩽normal | 17 (36%) | 64% |
| Unknown | 1 (2%) | |

*Clinical course*

Eighteen patients (36%) achieved complete remission without persisting residual mass. Ten patients (21%) with partial response >50% were considered as complete responders with persisting residual mass. Five other patients (11%) achieved a response ⩾50%. Two patients had stable disease, and 13 have died during treatment. Tumoral response was comparable in kidney and elsewhere in the 38 evaluable patients. The seven patients with partial response or stable disease died from disease progression.

Two of the 28 complete responders died without evidence of progressive disease at 3 and 4 months after CR achievement (sudden death, one patient; probable pulmonary thromboembolism, one patient). Ten of the 28 complete responders relapsed 3–40 months after CR achievement. Estimated 4 year disease-free survival was 58 ± 9%. The kidney was involved at relapse in four patients. Three patients died shortly after relapse. Five patients were alive with relapse and two patients were alive in second partial response at the closing date.

So far 25 patients have died. With a median follow-up of 12 months, actuarial overall survival was estimated to be 46 ± 7% at 48 months (Figure 1).

*Toxicity*

Forty of our 48 patients received their first chemotherapy cycle without dosage modification. Only one of the three patients with marked renal impairment had dose reduction of chemotherapy. His renal function could not be restored and he died. The two other patients received full-dose chemotherapy and their renal function was restored. In ten other patients with initial renal impairment renal function was restored as assessed by their serum blood urea nitrogen (BUN) and creatinine levels at the end of the induction regimen.

**Table II** Characteristics of lymphomatous involvement of the kidney

| | *Total number of patients* | *Single nodule* | *Multiple nodules* | *Diffuse infiltration* | *Perirenal mass* |
|---|---|---|---|---|---|
| Total no. of patients | 48 | 10 | 15 | 9 | 14 |
| Bilateral infiltration | | | | | |
| Yes | 21 | 1 | 14 | 3 | 3 |
| No | 27 | 9 | 1 | 6 | 11 |
| Perinephric space | | | | | |
| Involved | 22 | 3 | 0 | 6 | 13 |
| Normal | 23 | 6 | 14 | 2 | 1 |
| Unknown | 3 | 1 | 1 | 1 | 0 |
| Renal hilum involvement | | | | | |
| Yes | 17 | 2 | 1 | 5 | 9 |
| No | 22 | 6 | 11 | 3 | 2 |
| Unknown | 9 | 2 | 3 | 1 | 3 |
| Para-aortic adenopathies | | | | | |
| Yes | 33 | 8 | 6 | 6 | 13 |
| No | 15 | 2 | 9 | 3 | 1 |
| Mesenteric adenopathies | | | | | |
| Yes | 19 | 3 | 5 | 3 | 8 |
| No | 26 | 7 | 9 | 6 | 4 |
| Unknown | 3 | 0 | 1 | 0 | 2 |
| Creatinine level ($\mu$mol $^{-1}$) | | | | | |
| >120 | 13 | 1 | 4 | 3 | 5 |
| ⩽120 | 35 | 9 | 11 | 6 | 9 |

**Table III** Haematological characteristics of lymphoma patients with renal involvement

| | *Total number of patients* | *Single nodule* | *Multiple nodules* | *Diffuse infiltration* | *Perirenal mass* |
|---|---|---|---|---|---|
| Total no. of patients | 48 | 10 | 15 | 9 | 14 |
| Age (years) | | | | | |
| >55 | 21 | 3 | 8 | 4 | 6 |
| ≥55 | 27 | 7 | 7 | 5 | 8 |
| Histology | | | | | |
| Diffuse large cell | 29 | 5 | 12 | 4 | 8 |
| Lymphoblastic | 4 | 0 | 2 | 1 | 1 |
| Others | 15 | 5 | 1 | 4 | 5 |
| Ann Arbor stage | | | | | |
| IE-IIE | 4 | 0 | 0 | 1 | 3 |
| IV | 44 | 10 | 15 | 8 | 11 |
| Performance status (ECOG) | | | | | |
| 0 or 1 | 28 | 8 | 11 | 2 | 7 |
| 2 | 10 | 2 | 2 | 3 | 3 |
| 3 or 4 | 10 | 0 | 2 | 4 | 4 |
| Tumoral mass (cm) | | | | | |
| ≥10 | 33 | 6 | 8 | 8 | 11 |
| <10 | 14 | 3 | 7 | 1 | 3 |
| Unknown | 1 | 1 | 0 | 0 | 0 |
| LDH level | | | | | |
| ≤normal | 16 | 5 | 4 | 1 | 6 |
| >normal | 31 | 4 | 11 | 8 | 8 |
| Unknown | 1 | 1 | 0 | 0 | 0 |
| Bone marrow involvement | | | | | |
| Yes | 15 | 2 | 4 | 4 | 5 |
| No | 32 | 7 | 11 | 5 | 9 |
| Unknown | 1 | 1 | 0 | 0 | 0 |

**Figure 1** Overall survival of the 48 patients with non-Hodgkin's lymphoma and renal involvement included in the LNH-84 or LNH-87 protocol.

Acute renal insufficiency developed in five of the 35 patients with initial serum creatinine level <120 µmol $l^{-1}$. The mechanism was known in only three patients: septic haemolysis in one patient, drug-induced toxicity in one patient and lethal acute tumour lysis in one patient. Long-term follow-up showed mild renal impairment in two patients 42 and 12 months after CR achievement. Pyelocalyceal dilation and retraction of renal contour was observed on CT scan in one patient each.

The pattern of other toxicities was similar to that expected in a population of patients in the LNH-84 study with similar age and performance status distribution. It was not influenced by impairment of renal function at diagnosis.

### *Prognostic factors*

The LNH-84 prognostic index identified a subgroup of 40 high-risk patients and a subgroup of eight intermediate-risk patients. International prognostic index identified a subgroup of two low-risk patients, seven low- to intermediate-risk patients, 19 high- to intermediate-risk patients and 20 high-risk patients.

Renal parameters associated with a high CR rate were (Table IV) normal perinephric space ($P = 0.01$), multiple nodular involvement of the kidney ($P = 0.02$) and normal kidney hilum ($P = 0.02$).

Two renal parameters had adverse prognostic significance for survival: renal hilum involvement ($P = 0.02$) and diffuse renal infiltration ($P = 0.01$). Only three other prognostic factors were indicators of long survival, namely performance status 0 or 1 ($P = 0.001$), normal LDH level ($P = 0.02$) and diffuse large-cell histological subtype ($P = 0.03$). A Cox model identified only two independent prognostic factors for survival, namely performance status 2 or more and tumour size ≥10 cm.

### Discussion

Our report demonstrates that a high complete response rate and 4 year estimated survival rate can be achieved in aggressive NHL with renal involvement with the LNH-84 or LNH-87 regimen.

Distribution of histological subtypes of patients with lymphomatous involvement of the kidney was not significantly different from that found in all patients enrolled in the LNH-84 protocol (Coiffier *et al.*, 1989). Our study excluded low-grade NHL. However, only 7% of NHLs with renal involvement are low-grade NHL (Glicklich *et al.*, 1986; Richards *et al.*, 1990).

In 26 patients, renal involvement was diagnosed on CT scan only. Radiological criteria are sufficient to diagnose a renal lymphomatous involvement (Richards *et al.*, 1990). CT scan usually reveals a homogeneous mass that shares the density of soft tissues, with a minimal enhancement after injection (Heiken *et al.*, 1983). Incidences of renal involvement subtypes in our patients seemed somewhat different from those of previous reports (Richmond *et al.*, 1962; Jafri *et al.*, 1982; Heiken *et al.*, 1983). However, these studies included only a small number of patients and the differences were not significant. Multiple nodules was the most common type (33-43%), followed by single nodule (13-22%) and direct spread from perirenal mass (11-28%). Diffuse infil-

Table IV Parameters influencing complete response rate

| | Total | Complete response | No complete response | P-value |
|---|---|---|---|---|
| Total no. of patients | 48 | 28 | 20 | |
| Renal localisation | | | | |
| Unique nodule | 10 | 5 | 5 | |
| Multiple nodules | 15 | 13 | 2 | |
| Diffuse infiltration | 9 | 2 | 7 | |
| Perirenal mass | 14 | 8 | 6 | 0.02 |
| Renal hilum adenopathy | | | | |
| Yes | 17 | 7 | 10 | |
| No | 22 | 18 | 4 | |
| Unknown | 9 | | | 0.02 |
| Perinephric space infiltration | | | | |
| Yes | 22 | 9 | 13 | |
| No | 23 | 19 | 4 | |
| Unknown | 3 | | | 0.01 |
| Bilateral infiltration | | | | |
| Yes | 21 | 15 | 6 | |
| No | 27 | 13 | 14 | NS |
| Lomboarotic adenopathies | | | | |
| Yes | 33 | 19 | 14 | |
| No | 15 | 9 | 6 | NS |
| Creatinine level ($\mu$mol $l^{-1}$) | | | | |
| $>120$ | 13 | 7 | 6 | |
| $\leqslant 120$ | 35 | 21 | 14 | NS |
| Histology | | | | |
| Diffuse large cell | 29 | 21 | 8 | |
| Lymphoblastic | 4 | 2 | 2 | |
| Others | 15 | 5 | 10 | NS |
| Age (years) | | | | |
| $<55$ | 21 | 14 | 7 | |
| $\geqslant 55$ | 27 | 14 | 13 | NS |

tration was slightly more frequent (17%) in our study than in others (6%). Indeed, we included diffuse unilateral involvement in the diffuse infiltration, while other reports restricted this type of involvement only to bilateral infiltration (Heiken et al., 1983). Perirenal space was involved in 38–61% of patients (Jafri et al., 1982; Heiken et al., 1983) and even more (95%) in a histological study (Hartman et al., 1982). Lomboaortic lymph nodes were involved in 61–100% of patients (Richards et al., 1990; Jafri et al., 1982; Heiken et al., 1983).

Our study suggests an association between renal involvement and mediastinum or bone involvement. These findings have already been reported (Perrone et al., 1986; Richards et al., 1990).

Twenty-seven per cent of our patients, 25% of patients of Richards et al. (1990) and six out of nine patients of Geffen et al. (1985) had increased creatinine level ($>120\,\mu$mol $l^{-1}$). We report three patients who presented with acute renal failure (ARF). All of them had diffuse bilateral infiltration. This association has been already described (Kanfer et al., 1976; Randolph et al., 1983; Glicklich et al., 1986; Koolen et al., 1988). The ACVB regimen is an intensive chemotherapy regimen that includes no major nephrotoxic drugs, especially no methotrexate, in the induction phase. Thus, we believe that, based on our findings and the report of Geffen et al. (1985), a patient with renal insufficiency caused by lymphomatous involvement of the kidneys could be treated promptly with this combination chemotherapy in full dose. Transient haemodialysis should eventually be useful.

Eighty-four per cent of the 48 patients received their first chemotherapy cycle wjthout dosage modification. Indeed, initial impaired renal function rapidly improved with chemotherapy. However, one patient with initial normal renal function developed acute tumour lysis syndrome. This metabolic disorder can occur in NHL patients with bulky disease (Flombaum, 1988). Haematological toxicity in the 48 patients with renal involvement was similar to that in the 737 patients who received the LNH-84 protocol (Coiffier et al., 1989). Furthermore, we found no influence of initial impairment of renal function on haematological toxicity. We found that treatment responses in kidneys and in other sites of disease were similar and simultaneous. Radiological disappearance was often rapid, within 1 week to 6 months (Randolph et al., 1983; Glicklich et al., 1986; Cadman et al., 1988; Koolen et al., 1988; Richards et al., 1990).

Few series have described the results of treatment in patients with aggressive lymphoma and renal involvement. Complete remission has already been reported in primary NHL (Betta et al., 1986) or in patients with acute renal failure (Randolph et al., 1983; Koolen et al., 1988) with a follow-up of more than 18 months. CR rate ranged from 44% to 69% in previous series (Geffen et al., 1985; Richards et al., 1990). CR persisted for more than 1 year in 38–54% of patients (Geffen et al., 1985; Richards et al., 1990). Relapse has occurred in 22–50% of CR patients (Geffen et al., 1985; Richards et al., 1990). Twenty-three per cent of patients died within 3–4 months after diagnosis, either from disease progression or from toxicity of the induction regimen (Geffen et al., 1985; Richards et al., 1990). The present report describes the results of intensive combination chemotherapy regimens in a large number of patients: The CR rate and the percentage of patients who died during the induction phase were estimated to be 57% and 27% respectively, and agree with previous findings. Four year disease-free survival and 4 year overall survival were estimated to be $58 \pm 9$ and $46 \pm 7$% respectively.

Among initial renal characteristics, only diffuse infiltration and hilus involvement had a poor prognostic value for survival in our series. In a previous report (Glicklich et al., 1986), only one out of six patients with diffuse bilateral involvement (and ARF) was alive at 2 years despite a doxorubicin-containing regimen. However, multivariate analysis demonstrated that no renal characteristics retained independent prognostic value for survival. This analysis suggests that only tumoral mass ⩾ 10 cm (in kidney or elsewhere) and performance status ⩾ 2 had independent poor prognostic value for survival. Furthermore, we found a large predominance of high-risk or high- to intermediate-risk patients according to the prognostic index of the LNH-84 protocol or the international prognostic index. Ann Arbor stage IV (Kanfer *et al.*, 1976; Randolph *et al.*, 1983; Geffen *et al.*, 1985; Glicklich *et al.*, 1986; Richards *et al.*, 1990) and other extranodal localisation (Richards *et al.*, 1990) were nearly constant in previous reports, as in ours. Thus renal involvement affected staging rarely. Geffen *et al.* (1985) found at least one unfavourable prognostic factor in all patients, and three or more unfavourable prognostic factors in seven out of nine patients (Geffen *et al.*, 1985). Finally, our survival estimate agrees with that expected in a series of patients with aggressive NHL and similar distribution of prognostic index (Coiffier *et al.*, 1991; Shipp *et al.*, 1993).

In conclusion, renal involvement is rare and often asymptomatic. Indeed, it usually occurs in the setting of disseminated disease, with other unfavourable prognostic factors. Systemic chemotherapy improves renal function rapidly. Responses to an intensive chemotherapy regimen are similar to those obtained in other patients with advanced-stage NHL with similar initial prognostic factors.

### References

ARMITAGE, P. (1971). *Statistical Methods in Medical Research*. Halstead Press: London.

BETTA, P.G., BOTTERO, G. & COSIMI, M.F. (1986). Primary renal lymphoma. *Eur. Urol.*, **12**, 352-354.

BOSLY, A., LEPAGE, E., COIFFIER, B., DUPRIEZ, B., HERBRECHT, R., FILLET, G., DIVINE, M., NOUVEL, C., TILLY, H., BORDESSOULE, D., GAULARD, P. & GISSELBRECHT, C. (1993). Alternating chemotherapy does not improve results in poor prognosis aggressive lymphomas. LNH87 protocol group 3: a GELA study (abstract). *Blood*, **82** (Suppl.), **530**, 136a.

CADMAN, P.J., LINDSELL, D.R.M. & GOLDING, S.J. (1988). An unusual appearance of renal lymphoma. *Clin. Radiol.*, **39**, 452-453.

CARBONE, P.P., KAPLAN, H.S., MUSSHOFF, K., SMITHERS, D.W. & TUBIANA, M. (1971). Report of the committee of Hodgkin's disease staging. *Cancer Res.*, **31**, 1860-1861.

COIFFIER, B., GISSELBRECHT, C., HERBRECHT, R., TILLY, H., BOSLY, A. & BROUSSE, N. (1989). A multicenter study of intensive chemotherapy in 737 patients with aggressive malignant lymphoma. *J. Clin. Oncol.*, **7**, 1018-1026.

COIFFIER, B., GISSELBRECHT, C., VOSE, J.M., TILLY, H., HERBRECHT, R., BOSLY, A. & ARMITAGE, J.O. (1991). Prognostic factors in aggressive malignant lymphomas: description and validation of a prognostic index that could identify patients requiring a more intensive therapy. *J. Clin. Oncol.*, **9**, 211-219.

COX, D.R. (1982). Regression models and life-tables (with discussion). *J. R. Stat. Soc. B*, **34**, 187-220.

FLOMBAUM, C.D. (1988). Acute renal failure and dialysis in cancer patients. *Crit. Care Clin.*, **4**, 61-79.

GEFFEN, D.B., FISHER, R.I., LONGO, D.L., YOUNG, R.C. & DE VITA, V. (1985). Renal involvement in diffuse aggressive lymphomas: results of treatment with combination therapy. *J. Clin. Oncol.*, **3**, 646-653.

GISSELBRECHT, C., BOSLY, A., COIFFIER, B., D'AGAY, M.F., DIEBOLD, J., LEPAGE, E., GAULARD, P., HAIOUN, C., HERBRECHT, R., TILLY, H. & REYES, F. (1991). LNH-87 protocol: present and future (abstract 4.01). *Nouv. Rev. Fr. Hematol.*, **33** (Suppl.), 135.

GLICKLICH, D., SUNG, M.W. & FREY, M. (1986). Renal failure due to lymphomatous infiltration of the kidneys. Report of 3 new cases and review of the literature. *Cancer*, **58**, 748-753.

HARTMAN, D.S., DAVIS, C.J., GOLDMAN, S.M., FRIEDMAN, A.C. & FRITZSCHE, P. (1982). Renal lymphoma: radiologic-pathologic correlation of 21 cases. *Radiology*, **144**, 759-766.

HEIKEN, J.P., GOLD, R.P., SCHNUR, M.J., KING, D.L., BASHIST, B. & GLAZER, H.S. (1983). Computed tomography of renal lymphoma with ultrasound correlation. *J. Comput. Assist. Tomogr.*, **7**, 245-250.

JAFRI, S.Z.H., BREE, R.L., AMENDOLA, M.A., GLAZER, G.M., SCHWAB, R.E., FRANCIS, I.R. & BORLAZA, G. (1982). CT of renal and perirenal non-Hodgkin's lymphoma. *AJR*, **138**, 1101-1105.

KANDEL, L.B., MCCULLOUGH, D.L., HARRISON, L.H., WOODRUFF, R.D., AHL, E.T. & MUNITZ, H.A. (1987). Primary renal lymphoma. Does it exist? *Cancer*, **60**, 386-391.

KANFER, A., VANDEWALLE, A., MOREL-MAROGER, L., FEINTUCH, M.J., SRAER, J.D. & ROLAND, J. (1976). Acute renal insufficiency due to lymphomatous infiltration of the kidneys. A report of 6 cases. *Cancer*, **38**, 2588-2592.

KOOLEN, M.I., SCHIPPER, O., LIEBERGEN, F.J.H.M., KURSTJENS, R.M.A., UNNICK, A.J.M. & BOGMAN, M.J.J.T. (1988). Non-Hodgkin's lymphoma with unique localization in the kidneys presenting with acute renal failure. *Clin. Nephrol.*, **29**, 41-46.

LALLI, A.F. (1969). Lymphoma and the urinary tract. *Radiology*, **93**, 1051-1054.

MANTEL, N. (1966). Evaluation of survival data and two new rank-order statistics arising in its consideration. *Cancer Chemother. Rep.*, **50**, 163-170.

MARTINEZ-MALDONADO, M. & RAMIREZ DE ARELLANO, G.A. (1966). Renal involvement in malignant lymphomas. A survey of 49 cases. *J. Urol.*, **45**, 485-488.

PERRONE, T., FRIZZERA, G. & ROSAI, J. (1986). Mediastinal diffuse large-cell lymphoma with sclerosis. A clinicopathologic study of 60 cases. *Am. J. Surg. Pathol.*, **10**, 176-191.

RANDOLPH, V.L., HALL, W. & BRAMSON, W. (1983). Renal failure due to lymphomatous infiltration of the kidneys. *Cancer*, **52**, 1120-1221.

RICHARDS, M.A., MOOTOOSAMY, I., REZNEK, R.H., WEBB, J.A.W. & LISTER, T.A. (1990). Renal involvement in patients with lymphoma: clinical and pathological features in 23 cases. *Haematol. Oncol.*, **8**, 105-110.

RICHMOND, J., SHERMAN, R.S., DIAMOND, H.D. & CRAVER, L.F. (1962). Renal lesions associated with malignant lymphomas. *Am. J. Med.*, **32**, 184-207.

SHIPP, M., HARRINGTON, D., ANDERSON, J., ARMITAGE, J., BONADONNA, G., BRITTINGER, G., CABANILLAS, F., CANELLOS, G., COIFFIER, B., CONNORS, J., COWAN, R., CROWTHER, D., ENGELHARD, M., FISHER, R., GISSELBRECHT, C., HORNING, S., LEPAGE, E., LISTER, A., MEERWALDT, J., MONTSERRAT, E., NISSEN, M., OKEN, M., PETERSON, B., TONDINI, C., VELASQUEZ, W. & YEAP, B. (1993). Development of a predictive model for aggressive lymphoma: The international NHL prognostic factors project. *N. Engl. J. Med.*, **329**, 987-994.

STRAUSS, D.J., FILIPPA, D.A., LIEBERMAN, P.H., KOZINER, B., TZVI THALER, H. & CLARKSON, B.D. (1983). The non-Hodgkin's lymphomas. I. A retrospective clinical and pathologic analysis of 499 cases diagnosed between 1958 and 1969. *Cancer*, **51**, 101-109.

THE NON-HODGKIN LYMPHOMA PATHOLOGIC CLASSIFICATION PROJECT (1982). National Cancer Institute sponsored study of classifications of non Hodgkin's lymphomas: a summary and description of a working formulation for clinical usage. *Cancer*, **49**, 2115-2135.

WENTZELL, R.A. & BERKHEISE, R. (1955). Malignant lymphomatosis of the kidneys. *J. Urol.*, **74**, 177-185.